# Feasibility of simplifying renal dosimetry in ^177^Lu peptide receptor radionuclide therapy

**DOI:** 10.1186/s40658-018-0210-2

**Published:** 2018-07-05

**Authors:** Anna Sundlöv, Johan Gustafsson, Gustav Brolin, Nadja Mortensen, Rebecca Hermann, Peter Bernhardt, Johanna Svensson, Michael Ljungberg, Jan Tennvall, Katarina Sjögreen Gleisner

**Affiliations:** 1 Oncology and Pathology, Department of Clinical Sciences Lund, Skåne University Hospital, Lund University, Lund, Sweden; 2 Department of Medical Radiation Physics, Lund University, Lund, Sweden; 3 Department of Oncology, Sahlgrenska University Hospital, Gothenburg, Sweden; 4 Department of Radiation Physics, University of Gothenburg, Gothenburg, Sweden; 5 Department of Medical Physics and Biomedical Engineering, Sahlgrenska University Hospital, Gothenburg, Sweden; 6 Department of Oncology, Skåne University Hospital, SE-221 85 Lund, Sweden

**Keywords:** PRRT, Dosimetry, Neuroendocrine, 177-Lutetium, Dotatate, SPECT

## Abstract

**Background:**

Recently, ^177^Lu-dotatate therapy for neuroendocrine tumours has received regulatory approval. Dosimetry can be used to optimize treatment on an individual basis, but there is no international consensus as to how it should be done.

The aim of this study is to determine a feasible and accurate dosimetry method to guide individualized peptide receptor radionuclide therapy (PRRT) for patients with neuroendocrine tumours.

As part of a clinical trial on ^177^Lu-dotatate therapy, renal dosimetry was performed for all patients in each treatment cycle, using a hybrid planar-SPECT/CT method. In the present study, we use the image data acquired from 22 patients and 119 cycles and define a set of alternative treatment planning strategies, each representing a simplification in terms of image acquisition and dosimetric calculations. The results from the simplified strategies are compared to the results from the protocol-prescribed hybrid planar-SPECT/CT-based method by analysing differences both in per-cycle and total cumulative absorbed dose (AD) analyses.

**Results:**

In general, the SPECT-based methods gave results that were largely consistent with the protocol-specified hybrid method, both in the per-cycle and cumulative AD analyses. Notably, performing one SPECT/CT per cycle at 96 h yielded ADs that were very similar to the protocol method. The methods using planar dosimetry resulted in larger variations, as expected, while giving 4 cycles to all patients resulted in the largest inter-individual differences in cumulative AD.

**Conclusions:**

Performing one SPECT/CT at 96 h in every treatment cycle gives sufficiently reliable dosimetric results to base individualized treatment planning on, with a reasonable demand on resources.

**Electronic supplementary material:**

The online version of this article (10.1186/s40658-018-0210-2) contains supplementary material, which is available to authorized users.

## Background

Peptide receptor radionuclide therapy (PRRT), an established treatment for neuroendocrine tumours (NET), has recently received regulatory approval based on the results from the randomized phase III trial NETTER-1 [1]. Further optimization of PRRT may be achieved not only through improved patient selection with the use of clinical criteria [2], imaging [3] and molecular analyses[4] but also through improving the way the treatment is planned and carried through. The optimization strategies differ from centre to centre and include, but are not limited to, combining PRRT with chemotherapy [5, 6], combining different radionuclides [7] and using dosimetry to personalize treatment [8], the latter being the method of choice by our groups.

In external beam radiotherapy and brachytherapy, it is standard procedure to individually plan treatment by calculating absorbed dose (AD) to both target (i.e. tumour) and organs at risk, with the aim of optimizing the AD delivery so as to achieve a high probability of anti-tumour effect with a low risk as reasonably achievable of serious toxicity. This concept can also be applied to systemic radiotherapy such as PRRT, although there is as of yet no international consensus on how this should be done.

Our groups have, over the past several years, systematically worked to improve methods and clinical applications in the realm of dosimetry-guided systemic radiotherapy, with the long-term aim of improving treatment planning and results [9–17]. As part of this development, we designed a phase II trial (NCT01456078, “Iluminet”) to investigate the feasibility, safety and efficacy of individualized PRRT based on patient selection and dosimetry. The trial has been approved by the institutional ethics committee (ref. no. 2011/287) and national regulatory authorities. Further details on the protocol can be found at www.clinicaltrials.gov. All patients have given their written informed consent to participate.

In the Iluminet trial, the hypothesis was that by using individualized treatment planning, the balance between treatment effect and toxicity is optimized. Patients with advanced, progressive NET were treated with 7400 MBq ^177^Lu-dotatate at 8–12-week intervals, and detailed post-therapeutic imaging and dosimetry were performed in all patients after every cycle. The number of cycles each patient received was determined by a combined evaluation of dosimetry, treatment effect and toxicity. Patients with risk factors for nephrotoxicity received treatment up to a cumulative renal biologically effective dose (BED) of (27 ± 2) Gy, and patients without such risk factors were offered to continue up to (40 ± 2) Gy. This individualized approach led to a wide range in the number of cycles per patient, differing substantially from the standard four treatment cycles [18].

The dosimetric method used per protocol in the Iluminet trial is a hybrid planar-SPECT/CT method, where the time-retention curve is derived from serial planar images, and the absorbed dose-rate information from a SPECT/CT is used for rescaling into a time-dose rate curve. Performing such dosimetry for each cycle is time-consuming both for the patients and for the therapeutic team of technologists, physicists and physicians. In the context of a prospective clinical trial exploring the feasibility of individualized, dosimetry-based treatment planning, it is justified to use such a complex method. However, if this concept is to be brought to clinical routine, it is of interest to determine whether simplifications can be made without substantially affecting the estimated ADs.

In the present study, we use the image data acquired during the trial and define a set of alternative treatment planning strategies, each representing a simplification in terms of image acquisition and dosimetric calculations. The alternative strategies include the current standard of 4 cycles to all patients, planar dosimetry, simplified hybrid dosimetry and dosimetry based on one SPECT/CT only. Additionally, we have applied the method proposed by Hänscheid et al. [19] of using one single measurement after 4 days for both planar and SPECT/CT data. The results from the simplified strategies are compared to the results from the protocol-prescribed hybrid planar-SPECT/CT-based method and are evaluated both in terms of per-cycle differences as well as the cumulative absorbed dose over all cycles.

Thus, the aim of the work presented in this paper is to investigate the ADs that are obtained when simplified treatment strategies or dosimetry schemes are used as alternatives to the more complex protocol-prescribed method, in order to make conclusions of possible simplifications for future dosimetry-based ^177^Lu-PRRT.

## Methods

The image data on which the present analyses are based include the same patients as in the previously published interim analysis of the Iluminet trial [18]. Of the 51 patients that had been included at the time of the interim analysis, 22 had completed treatment as planned, i.e. had reached the targeted BED to the kidneys. It is on this subset of patients that the present analyses have been performed, comprising dosimetry of 119 cycles in total.

### Patient image acquisitions

The clinical trial protocol included patient imaging and kidney dosimetry in each cycle. Four anterior-posterior (AP) whole-body images were acquired at nominal times of 1, 24, 48 or 96 h (depending on which weekday the patient received treatment), and 168 h post administration, and one SPECT/CT study was performed at 24 h. An X-ray scout image was also acquired at 24 h to serve as attenuation map for whole-body image activity quantification.

Being a two-centre study, acquisitions were performed using a variety of camera systems (see Table 1. For whole-body scans, the matrix size was 1024 × 256 and the pixel size 2.2 × 2.2 mm^2^. Acquisition times were 10 or 20 min for the image at 1 h and 20 min for the other time points. SPECT projections were acquired using 60 projections over 360°, each of 45 s. The matrix size was 128 × 128 and the pixel dimensions approximately 4 × 4 mm^2^. For each of the gamma camera systems, the calibration factor was determined by measuring the system sensitivity for ^177^Lu in air, following established procedures [20], i.e. the count rate obtained in a planar image in response to a thin layer of ^177^Lu-solution placed in a Petri dish. The same calibration method was used for planar and SPECT images. Cross-calibration and traceability of the activity-meter readings at the two centres had previously been established using a source of ^177^Lu with activity statement from the National Physical Laboratory, UK. CT image acquisitions were performed using 120 kV and a low-dose protocol with either 2 mA or a current setting based on a noise index.

**Table 1:** Summary of the key characteristics for each of the camera systems, which were taken into account for the quantitative SPECT reconstruction, as well as the planar image quantification, used in the protocol. Medium-energy general purpose collimators were employed for all camera systems, and an energy window centred over the 208-keV photo peak

Camera	1	2	3	4	5	6	7	8
Model	GE Discovery VG	GE Discovery NM/CT 670	GE Millenium VG	GE Infinia	Philips IRIX	GE Discovery NM/CT 670	GE Discovery NM/CT 670 Pro	GE Discovery NM/CT 670 Pro
Crystal thickness (inches)	8/8	5/8	5/8	3/8	6/8	5/8	5/8	5/8
System sensitivity in 208 keV window (cps/MBq)	9.12	7.90	7.35	6.58	12.92	8.72	7.00	7.80
Energy window centred at 208 keV	20%	15%	20%	20%	20%	20%	20%	20%

### Quantification of activity, AD and BED

**Fig. 1:**
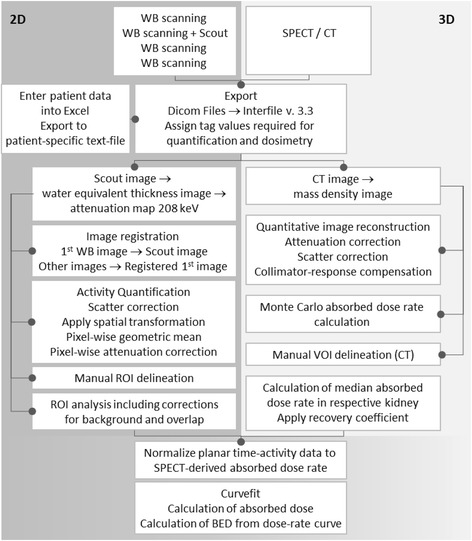
Schematic representation of the dosimetric method, implemented as part of LundADose and used per protocol (Iluminet trial)

The procedure for image-based dosimetry is summarized in Fig. 1.

The dosimetry calculations were performed using a program package called LundADose, implemented in the IDL programming language (Harris Geospatial Solutions, Broomfield, CO, USA). Prior to the actual computations, patient images were exported from the respective gamma camera systems, in DICOM image format, to the Interfile 3.3 format used internally in LundADose.

**Fig. 2:**
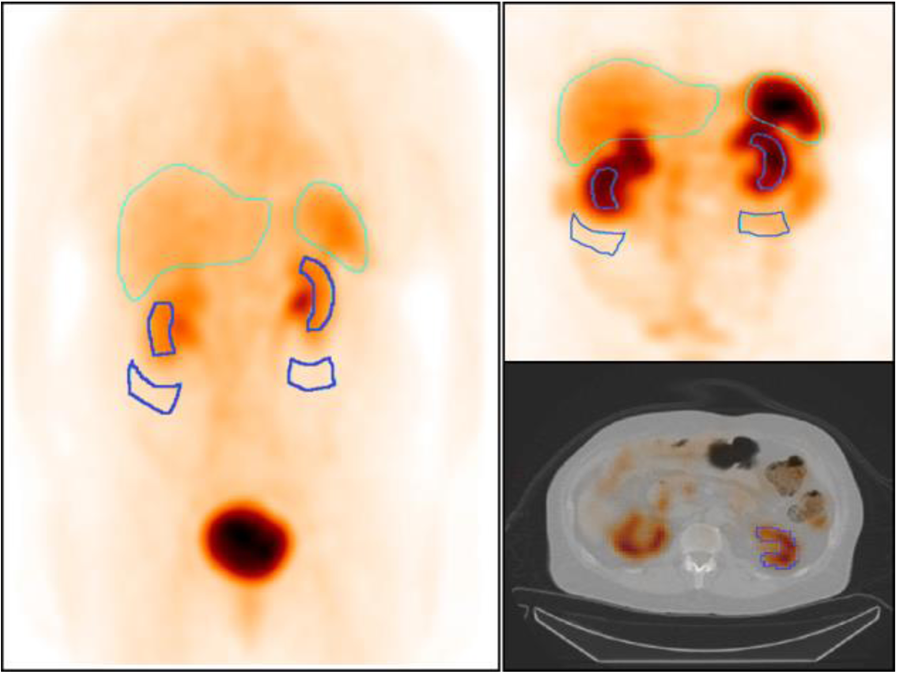
Examples of quantified planar images acquired 1 and 24 h p.i., with ROIs over kidneys and background (dark blue lines), as well as the SPECT/CT acquired 24 h p.i. with a VOI over the left kidney

The planar images were processed using a pixel-based implementation of the conjugate-view method, as previously reported [21, 22]. In the processed whole-body images from the four time points, regions of interest (ROIs) over the left and right kidneys were delineated, as well as a background ROI below each kidney (Fig. 2). Since the main purpose of the planar-based values was to determine the shape of the renal time-activity curve rather than the total kidney-activity content, it was not necessary that the ROIs encompassed the whole kidneys. Instead, care was taken to avoid overlapping tissues such as the liver or tumours. In cases when one kidney was severely overlapped by extra-renal activity uptakes, the effective half-time from the least overlapped kidney (often left) was used as an estimate also for the contralateral kidney. Background correction was accomplished by determining the activity per pixel in the respective background ROIs and then scaling to the number of pixels in the kidney ROIs, as well as to an estimated thickness of the background compartment within the kidney ROI [22].

Voxel-wise SPECT activity quantification was achieved as part of an iterative ordered-subsets expectation maximization image reconstruction, including corrections for attenuation and scatter, using a model-based method [23] and collimator response, and employing eight iterations and ten subsets. In order to use the CT image for attenuation and scatter corrections, the CT image values were first converted to mass density using a calibration-phantom-based relationship, as previously described [21]. From the density map, an attenuation map was generated by multiplication to mass attenuation coefficients valid for the soft tissue and cortical bone, for 208 keV. The quantitative SPECT image and the CT-derived mass-density image were used as basis for a Monte Carlo voxel-based dose-rate calculation using the EGS4/PRESTA algorithm [24]. Volumes of interest (VOIs) were drawn, by specialized technologists, along the kidney contour in the SPECT/CT images, mainly by guidance from the transversal CT. The VOIs were applied to the 3D absorbed-dose rate images, and the median absorbed-dose rate in the VOI was determined. This value was then divided by a recovery factor of 0.85, which had been established in a previous study where manual segmentation was performed by several operators in Monte Carlo-simulated SPECT/CT images [25]. This value of 0.85 was thus considered valid for all patient kidneys using the chosen methodology for image segmentation and SPECT image reconstruction.

A series of time-dose rate values at different times, *R*(*t*), were derived by combining the SPECT-derived absorbed-dose rate acquired at *t*_S_, i.e. *R*(*t*_S_), with the planar-derived activity values from different time points, *A*_P_(*t*), according to

1 $$ R(t)=\frac{A_{\mathrm{P}}(t)}{A_{\mathrm{P}}\left({t}_{\mathrm{S}}\right)}\cdotp R\left({t}_{\mathrm{S}}\right) $$

A curve, consisting of an initial straight line followed by a trailing mono-exponential function, was fitted to time-dose rate values. The mono-exponential function was fitted to the three last data points using weighted least squares, and the initial line was then determined analytically based on the values of the mono-exponential function at the time of the second data point and the first data point. This curve shape, in particular the initial straight line (equivalent to a trapezoid integration), was used as the simplest approach, realizing that the true, underlying renal time-activity curve, including the very fast initial redistribution [16, 26], was not resolvable from the two data points acquired during the first 24 h. The AD was determined as the area under the curve, calculated by analytical integration. Finally, the absorbed-dose rate curve was used as input to the calculation of the BED using a convolution-based method [12] with radiobiologic parameters *α*/*β* of 2.6 Gy and a repair half-time of 2.8 h [27].

The decision of whether the patient was to receive further treatment cycles was based on an overall evaluation of the cumulated renal BED, the projected renal BED in case of further treatment in relation to the target BED (27 or 40 Gy) as well as tumour effect and toxicity of treatment thus far.

### Simplified treatment strategies and dosimetry methods

Nine different, alternative treatment planning strategies were defined (see summary in Table 2). The hybrid dosimetry method used in the Iluminet trial is referred to as method P (for protocol) and is described in detail above.

A. *Four cycles to all patients*. The current standard treatment regimen, without patient-specific tailoring. For each patient and each cycle, the left-right mean renal AD was determined using method P, and the data for the four first cycles were summed. For patients who had only been given 3 cycles, the AD of a fourth cycle was set equal to that of the third cycle.
B. *Planar-based dosimetry with four whole-body acquisitions during the first cycle only*. The conjugate-view method was used to calculate the renal activity from the anterior-posterior planar images. A more typical implementation was here used such that ROIs encompassing the whole kidneys were delineated in the acquired count images, as well as background ROIs to subtract activity contributions from background tissues. In some cases, where the right kidney was extensively overlapped by the liver, the effective half-time estimated for the left kidney was used as a surrogate. The kidney mass was estimated by delineation in a CT image, and the dose-factor values (DFs) retrieved from OLINDA/EXM [28]. The left-right mean renal AD was determined for the first cycle. For the following cycles, it was assumed that the AD per administered activity remained unchanged. The AD for cycle *n* was calculated by multiplication to the ratios of administered activities, thus also taking small deviations from the nominal 7400 MBq into account, according to

**Table 2:** Summary of the imaging protocols for the dosimetry used in the Iluminet trial (P) and each of the alternative treatment planning strategies tested in the current analysis (A–I) (for further details, see the main text)

Treatment planning strategy	Description	Planar imaging	SPECT	Individualized, dosimetry-based treatment (yes/no)
Method P (as per clinical trial protocol)	Hybrid dosimetry in each cycle	Whole-body scintigraphy at 1, 24, 48 *or* 96 h and 168 h	1 SPECT at 24 h	Yes
Method A	4 cycles to all patients	Whole-body scintigraphy at 1, 24, 48 *or* 96 h and 168 h	1 SPECT at 24 h	No
Method B	Planar-based dosimetry 1st cycle only	Whole-body scintigraphy at 1, 24, 48 *or* 96 h and 168 h, 1st cycle only	None	Yes
Method C	Planar-based dosimetry in each cycle	Whole-body scintigraphy at 1, 24, 48 *or* 96 h and 168 h	None	Yes
Method D	Hybrid dosimetry 1st cycle only	Whole-body scintigraphy at 1, 24, 48 *or* 96 h and 168 h, 1st cycle only	1 SPECT at 24 h, 1st cycle only	Yes
Method E	Hybrid dosimetry 1st cycle + 1 SPECT/cycle thereafter	Whole-body scintigraphy at 1, 24, 48 *or* 96 h and 168 h, 1st cycle only	1 SPECT at 24 h	Yes
Method F	1 SPECT/cycle, identical effective halftime (51.6 h) assumed	None	1 SPECT at 24 h	Yes
Method G	1 SPECT/cycle at 96 h	None	1 SPECT at 96 h	Yes
Method H	1 SPECT at 96 h 1st cycle only	None	1 SPECT at 96 h, 1st cycle only	Yes
Method I	1 planar image at 96 h in each cycle	Whole-body scintigraphy at 96 h	None	Yes

5 $$ {D}_n={D}_1\frac{A_{\mathrm{inj},n}}{A_{\mathrm{inj},1}} $$

C. *Planar-based dosimetry with four whole-body acquisitions in each cycle*. Planar image quantification was applied as in B. For each of the treatment cycles, the left-right mean renal AD was taken and the cumulative AD was calculated as the sum of the ADs/cycle.
D. *Hybrid dosimetry in the first cycle only, i.e. four whole-body acquisitions and one SPECT at 24 h*. The ADs for the following cycles were calculated using Eq. 5.
E. *Hybrid dosimetry in the first cycle, and one SPECT/CT at 24 h each cycle thereafter*. The AD contribution for cycle *n* was here calculated by multiplication of the AD for cycle 1, to the ratios of SPECT-derived absorbed dose rates. The AD was determined as the mean of the left and right kidney ADs. For instance, the AD for the left kidney and cycle *n*, *D*_L, *n*_, was obtained using

6 $$ {D}_{\mathrm{L},n}={D}_{\mathrm{L},1}\ \frac{\ {R}_{\mathrm{L},n}\left({t}_{\mathrm{S},n}\right)}{R_{\mathrm{L},1}\left({t}_{\mathrm{S},1}\right)}\ \exp \left({\lambda}_{\mathrm{L},1}\ \left[{t}_{\mathrm{S},n}-{t}_{\mathrm{S},1}\right]\right) $$

The exponential function was here included to take into account possible differences in the time of the SPECT/CT acquisition for the different cycles, where the effective decay constant, *λ*_L, 1_, was obtained from the first cycle.

F. *One SPECT/CT at 24 h in each cycle, identical effective half-time for all patients assumed*. The shape of the time-activity curve was assumed to be identical and mono-exponential for all patients and was described by a decay constant, *λ*_ref_, based on the mean effective half-time of 51.6 h obtained in a previous study [18]. Also in this case, the AD for cycle *n* was determined as the mean of the left and right kidney ADs, where, for instance, for the left kidney, the AD was calculated as

7 $$ {D}_{\mathrm{L},n}=\kern0.5em \frac{\ {R}_{\mathrm{L},n}\left({t}_{\mathrm{S},n}\right)}{\lambda_{\mathrm{ref}}}\ \exp \left({\lambda}_{\mathrm{ref}}\ {t}_{\mathrm{S},n}\right) $$

G. *One SPECT/CT at 96 h in each cycle*. It was considered of interest to evaluate the simplification of using one SPECT/CT acquired at 4 days, as suggested in a recent publication [19]. Since the SPECT/CT was in most of our patients acquired on day 1, the absorbed-dose rate at 96 h was estimated by extrapolation using the effective half-time of the individual patient and kidney. The AD for cycle *n* was determined as the mean of the left and right kidney ADs, where, for instance, for the left kidney, the AD was calculated according to

8 $$ {D}_{\mathrm{L},n}=\frac{2\cdotp {t}_{\mathrm{ref}}}{\ln (2)}\ {R}_{\mathrm{L},n}\left({t}_{\mathrm{S},n}\right)\ \exp \left({\lambda}_{\mathrm{L},n}\ \left[{t}_{\mathrm{S},n}-{t}_{\mathrm{ref}}\right]\right) $$

where *t*_ref_ was thus 96 h [19].

H. *One SPECT/CT at 96 h in the first cycle only*. The AD for the first cycle was estimated as in alternative G, and for the following cycles using Eq. 5.
I. *One planar image at 96 h in each cycle*. The AD was estimated as in alternative G, but the factor *R* was obtained from planar images rather than SPECT.

### Data analysis

Systematic and random differences in the estimated AD per cycle between methods B to I and the protocol method P were investigated using Bland-Altman analysis [29]. The relative difference in AD between the alternative method and the reference method was compared to the mean AD of the two methods. The standard deviation (SD) was estimated based on a one-way analysis of variance [30], i.e. the variation of differences in estimated AD was assumed to be the result of a combination of inter- and intra-patient variability. Results were summarized quantitatively in terms of the Limits of Agreement (LOAs) that were defined as the average difference ± 2SD. Method A was not included in the Bland-Altman analysis as the AD/cycle used was in this case determined using the reference method (P).

The effect of the different treatment planning strategies on the final cumulative AD was also analysed. For each patient, the left-right mean renal AD was calculated for each cycle and then summed over the actually given number of cycles. For method A, the ADs for the first 4 cycles given were summed. The cumulative AD for each alternative method was set in relation to the AD for the protocol-specified dosimetry method. In order to be able to conclude which methods would have given an equivalent cumulative AD, an analysis of the uncertainty in the AD/cycle for the protocol method was done (see Additional file 1), giving an estimated fractional uncertainty of 10% (1SD). The uncertainty in the cumulative AD from multiple cycles was calculated using the law of propagation of uncertainty [31]. As information on the covariance in the AD estimates between cycles was not available, it was not considered in the uncertainty propagation. The confidence interval (CI) of the protocol-specified dosimetry was estimated as the cumulative absorbed dose ± 2SD.

Since method G was considered of particular interest (see the “Results” section) and we had access to SPECT/CT in six patients at four time points, a small additional analysis was made in which the AD resulting from method G was compared to the AD determined from four SPECTs/CT (see Additional file 1).

## Results

### Per-cycle analysis

**Fig. 3:**
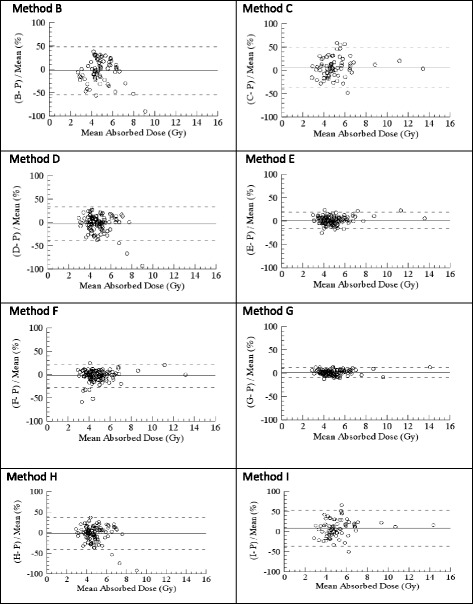
Pairwise quantification (Bland-Altman plots) of the limits of agreement (LOAs, dashed lines) of the relative AD difference for the alternative dosimetry protocols (B–I). The AD/cycle from each method is compared to that of method P (hybrid dosimetry in every treatment cycle, as per protocol) and normalized to the mean of the two methods. **B**: Planar dosimetry first cycle only (2SD = 51%). **C**: Planar dosimetry in all cycles (2SD = 43%). **D**: Hybrid dosimetry first cycle only (2SD = 36%). **E**: Hybrid dosimetry cycle one, and one SPECT/CT at 24 h in the following cycles (2SD = 17%). **F**: One SPECT/CT at 24 h in each cycle and constant half-time (2SD = 25%). Methods G, H and I are all based on Eq. 8 (see the “Methods” section). **G**: One SPECT/CT at 96 h in all cycles (2SD = 11%). **H**: One SPECT/CT at 96 h first cycle only (2SD = 39%). **I**: Planar dosimetry at 96 h in all cycles (2SD = 45%)

The results of the Bland-Altman analysis of the per-cycle AD of the alternative dosimetry methods B–I vs the protocol-specified dosimetry method are shown in Fig. 3. None of the methods studied resulted in marked systematic differences in the mean AD/cycle. The planar-based methods (B, C and I) resulted in the largest random differences with LOAs of − 3 ± 51%, 6 ± 43% and 8 ± 45%, respectively. Performing dosimetry in the first cycle and none thereafter also yielded wide LOAs of − 3 ± 36% with method D (hybrid dosimetry method) and − 2 ± 39% when applying Eq. 8 (method H). Using method F, i.e. one SPECT/cycle at 24 h, and a constant value of the effective half-time (51.6 h), the LOAs were − 2 ± 25%, while when using SPECT/CT hybrid dosimetry in the first treatment cycle and one SPECT/cycle in subsequent cycles (method E), LOAs were 1 ± 17%. The method whose results were most consistent with those achieved using the protocol-specified dosimetry method was method G (one SPECT/cycle at 96 h and application of Eq. 8) resulting in LOAs of 1 ± 11%.

### Cumulative absorbed dose analysis

Results of the comparisons of the total cumulative AD with methods A–I vs the AD achieved with the protocol-specified dosimetry method (± 2SD) are shown in Table 3. Giving 4 cycles to all patients (method A) yielded ADs that were consistent with dosimetry-based treatment in 4/22 (18%) patients, i.e. there were four patients who received 4 cycles when treated according to protocol. Planar dosimetry first cycle only (method B) and in all cycles (C and I) were consistent with method P in 3/14 (21%), 6/14 (43%) and 4/14 (29%) patients, respectively. SPECT-based dosimetry first cycle only (methods D and H) yielded consistency in 11/22 (50%) and 7/22 (32%) patients, respectively. The methods including SPECT in each cycle (E, F and G) were consistent with the reference ADs in 20 (91%), 19 (86%) and 22 (100%) of the 22 patients, respectively.

**Table 3:**
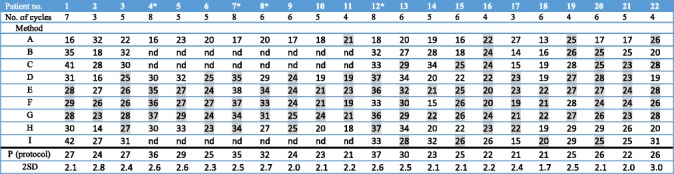
The cumulative AD over all cycles that would have been reached using alternative treatment strategy A (4 cycles to all) or estimated using alternative dosimetric methods (B–I) given the same no. of cycles as per protocol. “nd” indicates that the AD was not determined by that method

*Patients treated in step 2 (renal BED to (40 ± 2) Gy)

Grey indicates values that are within the estimated confidence interval for method P (AD for P ± 2SD). B: Planar dosimetry first cycle only. C: Planar dosimetry all cycles. D: Hybrid dosimetry first cycle only. E: Hybrid dosimetry first cycle and one SPECT/CT at 24 h in the following cycles. F: One SPECT/CT at 24 h in each cycle. G: One SPECT/CT at 96 h in all cycles. H: One SPECT/CT at 96 h first cycle only. I: Planar dosimetry at 96 h in all cycles. “No. of cycles”: the number of cycles actually given, based on method P

The practical consequences of using one method or another can be illustrated by a few patient examples: patient 1 received seven treatment cycles (27 Gy) in the protocol. Had we used planar dosimetry (method B or C), we would have overestimated the dose/cycle and interrupted treatment prematurely. Had the patient received only 4 cycles, the cumulative renal AD would have been 16 Gy. Patient 2 had an obstructive nephrolithiasis shortly after receiving cycle 2 resulting in a very high AD to the left kidney. In such a case, an estimated AD based on cycle 1 (methods B and D) would have underestimated the actual AD delivered. Hypothetically, if treatment would have been given based on method B or D, the patient would have received another 1 to 2 cycles resulting in an extrapolated total renal AD of 30 to 40 Gy, potentially causing further damage to the patient’s kidneys. Dosimetry in each cycle is helpful in determining a safe number of cycles for the continued treatment in such cases. Patient 14 is also an example of deviation from the expected in the sense that the half-time differed from the mean to such an extent that method F, which in most cases gave an acceptable result, would have led to a considerable underestimation of the AD/cycle. On the other hand, using method G in all patients would have reduced the number of imaging time points by 75% (once instead of four times) and the time spent for imaging by approximately 67% (from 120 to 40 min). From the small comparative analysis with four SPECTs/CT per cycle (Additional file 1), method G yielded deviations comparable to those obtained using the hybrid method, with a maximum deviation of − 11%.

## Discussion

The overall aim in this study was to elucidate whether it is possible to use a less demanding treatment strategy or dosimetry method in individualized ^177^Lu-dotatate therapy, as compared to that used in the ongoing clinical trial (Iluminet), while maintaining a similar degree of accuracy in renal AD estimates.

For the clinical trial protocol, a hybrid dosimetry method has been used. This choice was a compromise between the axial coverage offered by the whole-body planar images, and the superior accuracy of SPECT/CT. Moreover, at the time of the writing of the clinical trial protocol, performing serial SPECTs in each cycle, including the subsequent processing and analysis of 3D images, was deemed too time-consuming to be feasible.

For the evaluation of the AD per cycle for the alternative treatment strategies (Fig. 3), we conclude that the dosimetry methods in which SPECT/CT are included are superior to the purely planar-based methods and that including one SPECT/CT in each cycle improves the dosimetric accuracy considerably. Results based on the cumulative AD (Table 2) are consistent with results from the per-cycle analysis. Dosimetry method G, including one SPECT/CT in each cycle at 96 h, yields results that are equivalent with the protocol dosimetry method P but with much lower demands on resources. Methods E and F, which also use SPECT in every cycle, give similar but slightly less consistent results.

**Fig. 4:**
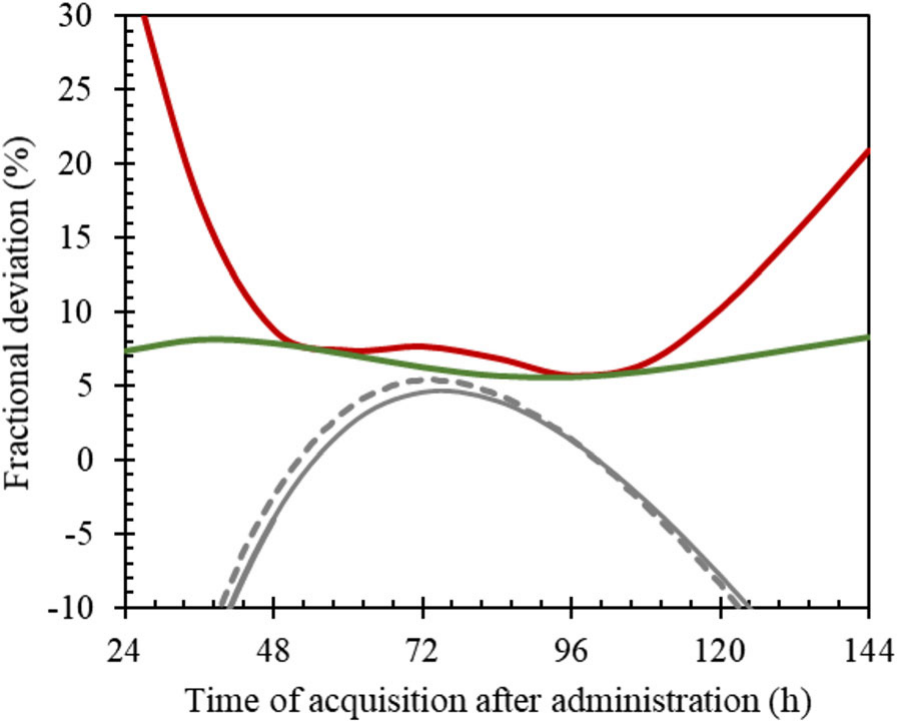
Fractional deviation between the actually delivered AD and the AD calculated using method G, as function of the time of acquisition for the single image (*t*_ref_ in Eq. 8). The dashed line shows the theoretical fractional deviation (*E*_1_), following Eqs. 9 and 10. Solid lines show deviations obtained for the patient data, determined as the mean value of *E*_2_ (grey), the SD around the mean *E*_2_ (green) and the root-mean square deviation in *E*_2_ (red)

Method G was implemented following the original work by Hänscheid et al. [19], where an approximate method for estimation of the area under the time-activity curve based on one single image acquisition was developed. The approximation can be derived from the expression of the absorbed dose for a mono-exponential curve, following

9 $$ D=\kern0.5em \frac{R\left({t}_{\mathrm{ref}}\right)\cdotp \exp \left(\lambda\ {t}_{\mathrm{ref}}\right)}{\lambda }=\frac{R\left({t}_{\mathrm{ref}}\right)\cdotp {2}^{\kern0.5em \left(\frac{t_{\mathrm{ref}}}{T_{1/2}}\right)}\cdotp {T}_{1/2}}{\ln (2)}\approx \frac{R\left({t}_{\mathrm{ref}}\right)\cdotp 2\cdotp {t}_{\mathrm{ref}}}{\ln (2)} $$

where the last, approximate equality is exact when the imaging time is equal to the effective half-time (*t*_ref_ = *T*_1/2_) or two times the effective half-time (*t*_ref_ = 2 *T*_1/2_). Hänscheid et al. [19] noted that the right-hand expression yields valid approximations when 0.75 *T*_1/2_ < *t*_ref_ < 2.5 *T*_1/2_ and in particular when *t*_ref_ = 96 h. If we denote the right-hand term as $\widehat{D},$ the theoretical fractional error, *E*_1_, becomes

10 $$ {E}_1=\frac{\left(\widehat{D}-D\right)}{D}=2f\cdotp {2}^{-f}-1 $$

with *f* = *t*_ref_/*T*_1/2_. Figure 4 shows the theoretical fractional error, *E*_1_, as a function of *t*_ref_ for an effective half-time 51.6 h, as obtained in a previous study [18]. It was considered of interest to evaluate the fractional error for patient data for different choices of *t*_ref_. Moreover, since in practice the imaging time point cannot be tuned to the effective half-life for a specific patient and cycle, for a given choice of *t*_ref_, the dispersion in effective half-times will translate into a variance in the fractional error. The data underlying results in Fig. 3 (method G) were thus reanalysed to a patient-based fractional error, *E*_2_ = (*D*_G_ − *D*_P_)/*D*_P_ , where *D*_G_ and *D*_P_ are the absorbed doses obtained from method G and P, respectively. Figure 4 shows the mean value of *E*_2_ obtained over all patients and cycles, the SD around the mean and the root-mean square deviation (RMSD) in *E*_2_, when the value of *t*_ref_ in Eq. 8 is varied between 24 and 144 h. It appears that the theoretical fractional error (*E*_1_) compares well with the fractional error obtained for patients (mean of *E*_2_) and that the overall deviation, as described by the RMSD, contains both systematic (mean of *E*_2_) and random components (SD of *E*_2_). When the dispersion in effective half-times is comparably modest, as for the kidney data analysed herein, the RMSD exhibits a valley when *T*_1/2_ < *t*_ref_ < 2*T*_1/2_ and is lowest near 96 h (*t*_ref_ ≈ 2*T*_1/2_). For an imaging time near 50 h (*t*_ref_ ≈ *T*_1/2_), the systematic component of the error is equally low as for 96 h, while the SD is higher, thus yielding a slightly higher RMSD. The RMSD is also more sensitive to the exact acquisition time at 50 h than at 96 h. Thus, acquisition at approximately 4 days appears to be a valid choice for this approximation. This is also confirmed by the uncertainty analysis based on SPECT/CT on day 4 (Additional file 1), where method G yields ADs that are equivalent to the protocol results. One drawback with our evaluation of method G is that the effective half-time estimated for the individual patient is included in the calculation of the absorbed-dose rate at 96 h since our SPECT/CTs were not acquired at 96 h. However, we still find it motivated to perform an independent evaluation of this new method, and the included implementation is deemed to be the fairest.

**Fig. 5:**
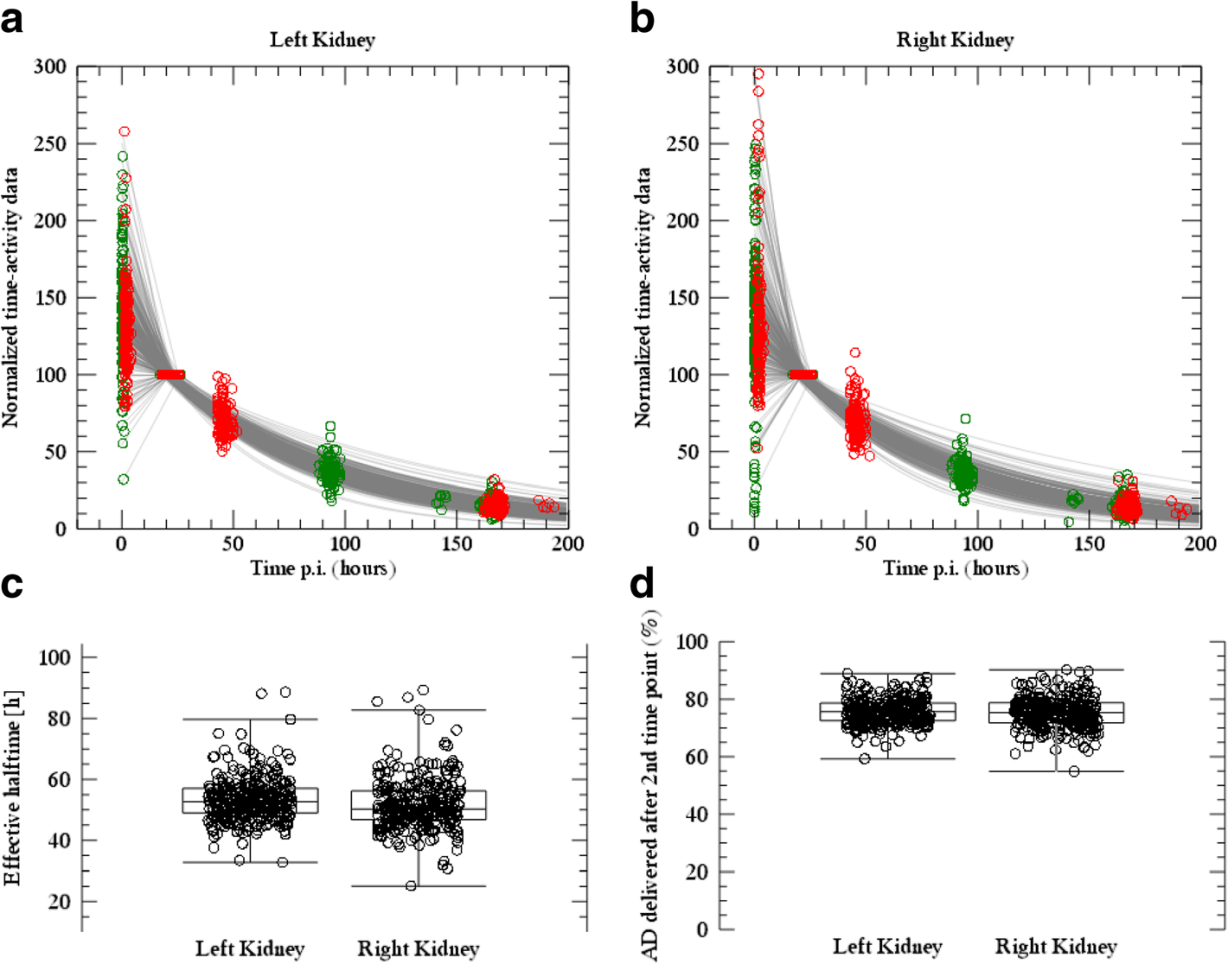
**a**, **b** Renal time-activity data (for the left and right kidneys, respectively) for 80 patients and all treatment cycles (in total approximately 300 curves), where curves have been normalized to a value of 100 at the second imaging time point (24 h). Red symbols are data acquired at one centre (39 patients), with nominal times of 1, 24, 48 and 168 h. Green symbols are acquired at the other centre (40 patients), with nominal times of 1, 24, 96 and 168 h. Grey lines are the fitted time-activity curves, also normalized to 100 at the time of the second imaging time point for each individual dataset. **c** Estimated effective half-times for the left and right kidneys after the second imaging time point. **d** The fractional absorbed dose that is due to activity retention after second imaging time point, determined as the absorbed dose delivered from 24 h and onwards, divided by the totally delivered absorbed dose

The comparably small deviations obtained using method F, i.e. one SPECT/CT for each cycle and a standard effective half-time for all patients, were unexpected and prompted further investigation. Figure 5 shows data derived from a larger patient material (80 subjects) than the one used for the analyses above. From this figure, we see that the shape of the time-activity curves after 24 h (A and B) as described by the effective half-time (C) is similar between patients, the median being 51.7 h. It is possible, however, that this uniformity in effective half-time is partly due to the uniformity of patient selection and procedures dictated by the clinical trial protocol. It would be of interest to perform a similar analysis in a real-life setting and in different PRRT centres. The vast spread in the first data point in the time-activity curve is a result of the imaging being performed at a time when there is a fast initial turnover and washout of ^177^Lu-dotatate via the kidneys [26]. This thus supports the choice of fitting function where the first data point, acquired at approximately 1 h, is decoupled from the fitting of the exponential tail and is thus not allowed to influence the assessment of the effective half-time. In spite of the vast spread observed, the AD using method F is in most cases consistent with the protocol dosimetry method P. This is explained by results in Fig. 5d where it is observed that the majority of the AD is delivered after the first 24 h (left kidney median 76% (interquartile range 73 and 79%), right kidney median 75% (interquartile range 72 and 79%)).

The conversion from activity to absorbed-dose rate is, according to the clinical protocol, performed using Monte Carlo-based radiation-transport calculation in a voxelized geometry. Since the range of the electrons emitted from ^177^Lu is shorter than the spatial resolution of the SPECT images, the radiation-transport calculation could possibly have been simplified to a multiplication by the assumption that the electron kinetic energy is absorbed locally in the voxel [24]. However, it was considered important not to neglect the photon contribution, since parts of the kidneys are located near organs with high uptake such as the spleen, and since in addition to gamma radiation, there are also low-energy X-rays emitted in the ^177^Lu decay that contribute to the self-absorbed dose. The Monte Carlo method was considered the best choice based on accuracy and availability. Notably, given that only one SPECT/CT is available, it is assumed that the fractional contribution from photons to the total absorbed energy in the kidneys is constant during the entire treatment cycle, whereas in reality, the photon contribution varies with time depending on the activity present in surrounding tissues.

When attempting a conclusion from these results, we can look at them from two different angles. From a methodological point of view, we want the most accurate method to be used, while from a clinical perspective, the question is how much accuracy is really needed in relation to the observed efficacy and toxicity. Of the methods compared in this analysis, dosimetry as per protocol is presumably the most accurate one (as the rest are simplifications of the same), but doing four SPECTs/CT in each cycle would probably yield even more accurate results although to the price of a more time-consuming procedure. Among the simplified methods analysed, we would choose the one with the smallest mean difference for each cycle and the narrowest LOAs.

The methods which best fit these criteria are E and G, both of which incorporate SPECT-based dosimetry in every cycle but are less resource-intense than the reference method P. The strategy that deviates the most is unequivocally method A, i.e. giving 4 cycles to all patients, followed by the planar-based dosimetry methods (B, C and I). This is consistent with what is already known about the drawbacks of planar dosimetry, namely the difficulties in taking into account individual variations in activity concentration, especially in tumours, the liver and intestines, which may then lead to over- or underestimation of the renal AD.

When choosing between methods E and G, one aspect to take into account is that for most PRRT centres, it would be more resource-consuming to perform a SPECT at 96 h since the patients would have to come back to the hospital. Whether or not to still go for method G then becomes a matter of whether or not the small difference seen in the Bland-Altman analyses (2SD of 11 and 17% for methods G and E, respectively) makes a relevant difference in real-life patient management.

From a clinical perspective, on the other hand, some would argue that to give 4 cycles to all patients is already a very effective treatment with a low degree of toxicity, so the need for further optimization is marginal. There are two objections to this: firstly, our obligation to know what we are doing when employing radiation for therapeutic purposes (analogous to the demands in other radiation therapy modalities), and secondly, when these patients progress months or years after their 4 cycles of PRRT, we would like to retreat them since the majority of the cases have not reached the limits for the organs at risk. Without initial dosimetric estimates, we have no grounds on which to plan further treatment.

Given the highly clinically and statistically significant effect of PRRT demonstrated in the NETTER-1 trial [1], together with the ample experience and long safety follow-up with the standard 4 cycles of treatment, perhaps the optimal treatment strategy is a mix between this and dosimetry-based treatment. This could be achieved by initially giving 4 cycles to all (or less if tolerance limits are reached before), but performing dosimetry as well. When the tumour later progresses, the patient can be re-treated to risk organ tolerance limits. In this manner, we get a good risk-benefit balance at each stage of the disease—early in the disease when the patient has a longer expected survival, we use a low-risk treatment, but once progression has been confirmed and the prognosis is another, it is more reasonable to assume the possible risks of higher ADs to risk organs associated with further treatment. Even with the relatively high renal BED limits used in the Iluminet trial, the toxicity has so far been limited (unpublished data), so it is still an open question which limits we should use in the future. Perhaps further retreatment beyond the limits used in this trial will be feasible for some patients under certain conditions. For this reason also, it will be of essence to incorporate dosimetry in PRRT planning, both in clinical routine and future clinical trials, and thereby progressively increase our understanding and body of knowledge.

## Conclusions

From a methodological and clinical point of view, we need an accurate dosimetric method demanding a reasonable amount of resources. Dosimetry based on one SPECT/cycle complies with these requirements, and it seems that performing the SPECT at 96 h gives reliable results.

## Additional file

Additional file 1: **Table S1.** Effective half-times and absorbed doses for six patients in which both four SPECT/CT and four planar whole-body scans were acquired. AD: absorbed dose, L/R mean: mean absorbed dose between the left and right kidneys, Diff: % difference for L/R mean absorbed dose values calculated as (Hybrid-SPECT)/SPECT. Table S2. Operator variability in estimated absorbed dose rate, effective half-time and absorbed dose. Op mean ± 1SD denotes the mean and standard deviation over the three operators, and L/R mean is the mean absorbed dose for the left and right kidneys. Upper rows: The notation of the three pharmacokinetic computer phantoms follow the notation used in (2). Uncertainty in absorbed dose is expressed as relative mean error (rE), relative standard deviation (rSD) and relative root-mean square error (rRMSE). Bottom rows: Results for patient studies with uncertainty expressed as the CV of the L/R mean. (DOCX 40 kb)

## Abbreviations

AD: Absorbed dose
AP: Anterior-posterior
BED: Biologically effective dose
CT: Computed tomography
LOAs: Limits of agreement
NET: Neuroendocrine tumour
PRRT: Peptide receptor radionuclide therapy
ROIs: Regions of interest
SD: Standard deviation
SPECT: Single-photon emission computed tomography
VOI: Volume of interest
