# Supplementary material for: Feasibility of simplifying renal dosimetry in 177Lu peptide receptor radionuclide therapy
Source: EJNMMI Phys. 2018 Jul 5;5:12. doi: 10.1186/s40658-018-0210-2 (PMC6031553; doi:10.1186/s40658-018-0210-2)
Supplement: Supplementary file 1 — Table S1. Effective half-times and absorbed doses for six patients in which both four SPECT/CT and four planar whole-body scans were acquired. AD: absorbed dose, L/R mean: mean absorbed dose between the left and right kidneys, Diff: % difference for L/R mean absorbed dose values calculated as (Hybrid-SPECT)/SPECT. Table S2. Operator variability in estimated absorbed dose rate, effective half-time and absorbed dose. Op mean ± 1SD denotes the mean and standard deviation over the three operators, and L/R mean is the mean absorbed dose for the left and right kidneys. Upper rows: The notation of the three pharmacokinetic computer phantoms follow the notation used in (2). Uncertainty in absorbed dose is expressed as relative mean error (rE), relative standard deviation (rSD) and relative root-mean square error (rRMSE). Bottom rows: Results for patient studies with uncertainty expressed as the CV of the L/R mean. (DOCX 40 kb) [file 40658_2018_210_MOESM1_ESM.docx]

## Supplemental Material for “Feasibility of simplifying renal dosimetry in ^177^Lu peptide receptor radionuclide therapy”

Anna Sundlöv^1^, Johan Gustafsson^2^, Gustav Brolin^2^, Nadja Mortensen^1^, Rebecca Hermann^4^, Peter Bernhardt^4,5^, Johanna Svensson^3^, Michael Ljungberg^2^, Jan Tennvall^1^, Katarina Sjögreen Gleisner^2^

^1^ Lund University, Skåne University Hospital, Department of Clinical Sciences Lund, Oncology and Pathology, Lund, Sweden

^2^ Department of Medical Radiation Physics, Lund University, Lund, Sweden

^3^ Department of Oncology, Sahlgrenska University Hospital, Gothenburg, Sweden

^4^ Department of Radiation Physics, University of Gothenburg, Gothenburg, Sweden

^5^ Department of Medical Physics and Biomedical Engineering, Sahlgrenska University Hospital, Gothenburg, Sweden

Correspondence to:

Anna Sundlöv, MD

Department of Oncology

Skåne University Hospital

SE-221 85 Lund

Sweden

E-mail: [anna.sundlov@med.lu.se](mailto:anna.sundlov@med.lu.se)

Tel: +46-46-175718

Orcid ID: 0000-0003-1565-3496

##

## Estimation of uncertainty in absorbed dose

The uncertainty in absorbed dose (AD) estimated using the hybrid planar-SPECT/CT based method was investigated in three different ways, using both patient and simulated image data. In addition, the uncertainty involved when using a single-time point SPECT/CT at day 4 [1] was assessed in patient data.

1. In six patients double acquisitions were performed, i.e. both planar and SPECT/CT imaging, at four time points. The AD obtained by the hybrid method was compared to that obtained from SPECT/CT. The difference in the left-right mean AD was calculated, and was regarded as a measure of the uncertainty contribution that arises when the curve shape is estimated from planar images instead of SPECT/CT. The AD calculated by the single-time point SPECT/CT at day 4 was also evaluated in this data set by comparison to the AD determined from four SPECT/CTs.
2. The inter-operator dependency, mainly due to the manual segmentation steps, was investigated in three patient studies. For each patient three technologists performed the calculations, including delineation of ROIs in planar, and VOIs in SPECT/CT images. For each patient study and each kidney this thus yielded three values of the effective half-life, absorbed-dose rate, and AD, for which the mean and standard deviation were calculated. The uncertainty in AD was summarized in terms of the coefficient of variation (CV), calculated from the left-right mean AD values as the standard deviation between the operators divided by the mean.
3. Three different voxel-based anthropomorphic models, coupled to pharmacokinetic (PK) models of 177Lu-DOTATATE were used, including reference values of the ADs in the left and right renal cortex and medulla for each of the phantoms [2]. Of note, the modelled pharmacokinetics did not aim for a similar left-right behaviour, and so left and right kidneys were in our evaluation regarded as separate test objects with different extent of overlap of liver and tumours. The anthropomorphic models were used as input to the SIMIND Monte Carlo program [3] for simulation of both planar whole-body AP scans and SPECT imaging, assuming imaging time-points as for the clinical procedure and settings as for camera 2 in Table 1. For each of the computer phantoms, three different technologists performed image segmentation in both planar and SPECT/CT images. The relative mean error, $rE,$ was determined according to

|  | $rE=\frac{\bar{D}}{D_{\mathrm{ref}}}-1$ | (S1) |
| --- | --- | --- |

where $\bar{D}$ was the mean AD for the three operators, and $D_{\mathrm{ref}}$ was the reference value for the particular phantom kidney. The relative standard deviation,$rSD$, was determined as the standard deviation in AD for the three operators, normalized to $D_{\mathrm{ref}}$. As a metric of both systematic and random uncertainties, the relative root-mean square error, $rRMSE$, was calculated according to

|  | $rRMSE=\frac{1}{D_{\mathrm{ref}}}\sqrt{\frac{1}{N}\sum_{i} \left( D_{i}-D_{\mathrm{ref}} \right)^{2}}$ | (S2) |
| --- | --- | --- |

#### Results

Table S1 shows dosimetric results for the six patients for whom both the hybrid method and a purely SPECT/CT-based method were used for calculation. In addition Table S1 includes ADs estimated using the single time-point SPECT/CT acquired at day 4.

Table S2 shows results for simulated data as well as the inter-operator variability estimated from patient data.

| **4 SPECT/CT** | | | | | | **Hybrid: 1SPECT/CT + 4 planar** | | | | | | **Single-time point SPECT/CT** | | | | |
| --- | --- | --- | --- | --- | --- | --- | --- | --- | --- | --- | --- | --- | --- | --- | --- | --- |
|  | **Eff half-time (h)** | | **AD (Gy)** | | | **Eff half-time (h)** | | **AD (Gy)** | | | **Diff** | **SPECT time (h)** | **AD (Gy)** | |  | **Diff** |
| **Pat.** | **Left** | **Right** | **Left** | **Right** | **L/R**  **mean** | **Left** | **Right** | **Left** | **Right** | **L/R mean** | **%** |  | **Left** | **Right** | **L/R mean** | **%** |
| 1 | 48 | 46 | 3.9 | 3.8 | 3.8 | 50 | 59 | 3.7 | 4.4 | 4.1 | 8 | 93 | 3.5 | 3.3 | 3.4 | -11 |
| 2 | 53 | 49 | 3.8 | 3.8 | 3.8 | 52 | 56 | 3.5 | 4.1 | 3.8 | 1 | 94 | 3.8 | 3.7 | 3.8 | -1 |
| 3 | 54 | 58 | 7.0 | 7.5 | 7.3 | 54 | 54 | 6.9 | 6.9 | 6.9 | -5 | 70 | 7.5 | 7.9 | 7.7 | 6 |
| 4 | 43 | 43 | 4.2 | 3.9 | 4.1 | 47 | 49 | 4.5 | 4.3 | 4.4 | 8 | 94 | 3.9 | 3.6 | 3.7 | -8 |
| 5 | 49 | 55 | 4.3 | 4.4 | 4.3 | 50 | 51 | 4.4 | 4.3 | 4.3 | 0 | 96 | 4.4 | 4.4 | 4.4 | 1 |
| 6 | 50 | 53 | 4.6 | 4.3 | 4.4 | 59 | 47 | 5.3 | 3.8 | 4.6 | 3 | 93 | 4.8 | 4.4 | 4.6 | 3 |

**Additional file 1: Table S1** Effective half-times and absorbed doses for six patients in which both four SPECT/CT and four planar whole-body scans were acquired. AD: absorbed dose, L/R mean: mean absorbed dose between left and right kidney, Diff: % difference for L/R mean absorbed dose values calculated as (Hybrid-SPECT)/SPECT.

|  | **AD rate (mGy/h)** | | **Eff half-time (h)** | | **AD (Gy)** | | | | **AD uncertainty (%)** | |
| --- | --- | --- | --- | --- | --- | --- | --- | --- | --- | --- |
|  | **Op mean ± 1 SD** | | **Op mean ± 1 SD** | | **Op mean ± 1 SD** | | **Reference** | | **rE ± rSD (rRMSE)** | |
|  | **Left** | **Right** | **Left** | **Right** | **Left** | **Right** | **Left** | **Right** | **Left** | **Right** |
| Phantom A | 35 ± 1 | 33 ± 0.5 | 48 ± 2 | 45 ± 3 | 3.3 ± 0.1 | 2.9 ± 0.1 | 3.51 | 3.44 | -7 ± 2 (7) | -15 ± 3 (15) |
| Phantom B | 31 ± 2 | 41 ± 1 | 49 ± 2 | 56 ± 4 | 3.0 ± 0.1 | 4.4 ± 0.2 | 3.05 | 4.02 | -2 ± 4 (4) | 9 ± 4 (10) |
| Phantom C | 42 ± 1 | 46 ± 1 | 54 ± 2 | 67 ± 5 | 4.2 ± 0.1 | 5.1 ± 0.1 | 4.13 | 4.78 | 2 ± 3 (3) | 6 ± 2 (6) |
|  | **Op mean ± 1 SD** | | **Op mean ± 1 SD** | | **Op mean ± 1 SD** | | |  |  |  |
|  | **Left** | **Right** | **Left** | **Right** | **Left** | **Right** | **L/R mean** |  | **CV (%)** |  |
| Patient 7 | 45 ± 4 | 45 ± 3 | 48 ± 3 | 44 ± 3 | 4.6 ± 0.3 | 4.2 ± 0.2 | 4.4 ± 0.05 | N/A | 1 | N/A |
| Patient 8 | 39 ± 2 | 39 ± 4 | 51 ± 2 | 48 ± 2 | 3.9 ± 0.2 | 3.8 ± 0.3 | 3.9 ± 0.3 | N/A | 7 | N/A |
| Patient 9 | 41 ± 3 | 45 ± 3 | 59 ± 1 | 53 ± 3 | 4.8 ± 0.3 | 4.7 ± 0.5 | 4.8 ± 0.4 | N/A | 8 | N/A |

**Additional file 2: Table S2** Operator variability in estimated absorbed dose rate, effective half-time and absorbed dose. Op mean ± 1 SD denotes the mean and standard deviation over the three operators, and L/R mean is the mean absorbed dose for left and right kidneys. Upper rows: The notation of the three pharmacokinetic computer phantoms follow the notation used in (2). Uncertainty in absorbed dose is expressed as relative mean error (rE), relative standard deviation (rSD) and relative root-mean square error (rRMSE). Bottom rows: Results for patient studies with uncertainty expressed as the CV of the L/R mean.

From Table S1 it is seen that the hybrid method yields slightly higher ADs than the purely SPECT-based method in four of the six patients, with a maximum deviation obtained of +8%. The uncertainty in renal AD using a purely SPECT/CT-based method was previously investigated by our group and obtained to ±6% (1 SD) [4], and when combining in quadrature this gives 10%. Table S1 also shows that the single-time point method based on one SPECT/CT yields deviations that are comparable to those obtained using the hybrid method, with a maximum deviation obtained of -11%.

From simulated images (Table S2, upper rows) the maximum rE obtained for the hybrid method is -15%, while the maximum rSD is 4%. The deviations are higher for the right kidney than the left, owing to a larger degree of overlap with liver and tumours in the planar images. Combining the rE and rSD gives a mean rRMSE of 8%, with a maximum of 15% obtained. Table S2, lower rows, shows that the inter-operator investigation in patients results in a maximum CV in AD of 8%.

When regarding results in tables S1 and S2 together, it appears reasonable to estimate the relative uncertainty in renal AD for one treatment cycle to approximately 10% (1 SD). This thus includes both uncertainty due to operator-dependent steps such as ROI- and VOI-drawing, and due to estimation of effective half-time from the planar images. One weakness in this analysis is the small number of measurements on which the estimation of the uncertainty is based. Moreover, it would be of interest to identify the separate contributions from the estimations of dose rate and the effective half-time, to the combined AD uncertainty. In that sense, this uncertainty analysis represent one first step, which should be complemented by future investigations.

#### References

1. Hanscheid H, Lapa C, Buck AK, Lassmann M, Werner RA. Dose Mapping after Endoradiotherapy with 177Lu-DOTATATE/-TOC by One Single Measurement after Four Days. J Nucl Med. 2017. doi:10.2967/jnumed.117.193706.

2. Brolin G, Gustafsson J, Ljungberg M, Gleisner KS. Pharmacokinetic digital phantoms for accuracy assessment of image-based dosimetry in (177)Lu-DOTATATE peptide receptor radionuclide therapy. Phys Med Biol. 2015;60:6131-49. doi:10.1088/0031-9155/60/15/6131.

3. Ljungberg M, Strand SE. A Monte Carlo program for the simulation of scintillation camera characteristics. Comput Methods Programs Biomed. 1989;29:257-72.

4. Gustafsson J, Brolin G, Cox M, Ljungberg M, Johansson L, Gleisner KS. Uncertainty propagation for SPECT/CT-based renal dosimetry in (177)Lu peptide receptor radionuclide therapy. Phys Med Biol. 2015;60:8329-46. doi:10.1088/0031-9155/60/21/8329.
